# Tetra­methyl­ammonium aqua­trichlorido­oxalatostannate(IV) monohydrate

**DOI:** 10.1107/S1600536813000895

**Published:** 2013-01-16

**Authors:** Yaya Sow, Libasse Diop, Kieran C. Molloy, Gabriele Kociok-Köhn

**Affiliations:** a Laboratoire de Chimie Minerale et Analytique (LACHIMIA), Departement de Chimie, Faculté des Sciences et Techniques, Université Cheikh Anta Diop, Dakar, Senegal; b Department of Chemistry, University of Bath, Bath BA2 7AY, England

## Abstract

The Sn^IV^ atom in the title compound, [(CH_3_)_4_N][Sn(C_2_O_4_)Cl_3_(H_2_O)]·H_2_O, obtained from the reaction between SnCl_4_ and [(CH_3_)_4_N]_2_C_2_O_4_·2H_2_O, is six-coordinated by three Cl atoms, an O atom of a water mol­ecule and two O atoms from an asymmetrically chelating oxalate anion. The environment around the Sn^IV^ atom is distorted octa­hedral. The anions are connected by the lattice water mol­ecule through O—H⋯O hydrogen bonds, leading to a layered structure parallel to (010). The cations are located between these layers and besides Coulombic forces are connected to the anionic layers through weak C—H⋯O and C—H⋯Cl inter­actions.

## Related literature

For background to halogentin(IV) chemistry, see: Hausen *et al.* (1986 ▶); Koutsantonis *et al.* (2003 ▶); Mahon *et al.* (2004 ▶); Patt-Siebel *et al.*(1986 ▶); Szymanska-Buzar *et al.* (2001 ▶); Tudela *et al.* (1986 ▶). For tin compounds containing an Sn—Cl bond in a *cis*- or *trans*-position, see: Fernandez *et al.* (2002 ▶); Hazell *et al.* (1998 ▶); Sow *et al.* (2010 ▶). For tin compounds containing carboxyl­ate moieties, see: Ng & Kumar Das (1993 ▶); Xu *et al.* (2003 ▶).
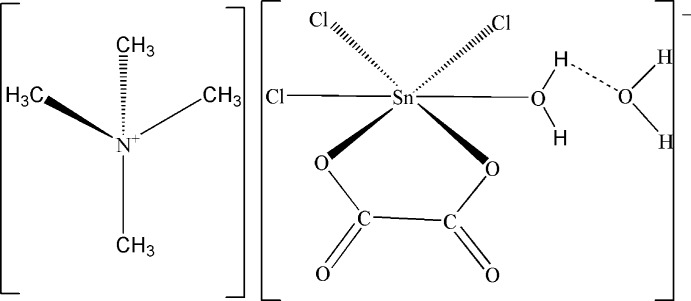


## Experimental

### 

#### Crystal data

(C_4_H_12_N)[Sn(C_2_O_4_)Cl_3_(H_2_O)]·H_2_O
*M* *_r_* = 423.24
Monoclinic, 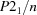

*a* = 7.2458 (1) Å
*b* = 22.2812 (2) Å
*c* = 9.6019 (1) Å
β = 98.015 (1)°
*V* = 1535.04 (3) Å^3^
*Z* = 4
Mo *K*α radiation
μ = 2.20 mm^−1^
*T* = 150 K
0.15 × 0.15 × 0.13 mm

#### Data collection

Nonius KappaCCD diffractometer
Absorption correction: multi-scan (*SORTAV*; Blessing, 1995 ▶) *T*_min_ = 0.734, *T*_max_ = 0.763
35849 measured reflections
4445 independent reflections
3855 reflections with *I* > 2σ(*I*)
*R*_int_ = 0.042

#### Refinement

*R*[*F* ^2^ > 2σ(*F* ^2^)] = 0.026
*wR*(*F* ^2^) = 0.062
*S* = 1.08
4445 reflections
175 parameters
4 restraints
H atoms treated by a mixture of independent and constrained refinement
Δρ_max_ = 0.92 e Å^−3^
Δρ_min_ = −0.79 e Å^−3^

### 

Data collection: *COLLECT* (Nonius, 1999 ▶); cell refinement: *DENZO* and *SCALEPACK* (Otwinowski & Minor, 1997 ▶); data reduction: *DENZO* and *SCALEPACK*; program(s) used to solve structure: *SIR97* (Altomare *et al.*, 1999 ▶); program(s) used to refine structure: *SHELXL97* (Sheldrick, 2008 ▶); molecular graphics: *ORTEP-3* (Farrugia, 2012 ▶); software used to prepare material for publication: *WinGX* (Farrugia, 2012 ▶).

## Supplementary Material

Crystal structure: contains datablock(s) I, global. DOI: 10.1107/S1600536813000895/wm2712sup1.cif

Structure factors: contains datablock(s) I. DOI: 10.1107/S1600536813000895/wm2712Isup2.hkl

Additional supplementary materials:  crystallographic information; 3D view; checkCIF report

## Figures and Tables

**Table 1:** Hydrogen-bond geometry (Å, °)

*D*—H⋯*A*	*D*—H	H⋯*A*	*D*⋯*A*	*D*—H⋯*A*
O5—H50*A*⋯O6	0.86 (2)	1.66 (2)	2.511 (2)	173 (3)
O5—H50*B*⋯O4^i^	0.85 (2)	1.78 (2)	2.6120 (19)	168 (3)
O6—H60*B*⋯O3^ii^	0.84 (2)	1.99 (2)	2.792 (2)	160 (3)
O6—H60*A*⋯O3^iii^	0.84 (2)	1.95 (2)	2.7840 (19)	172 (3)
O6—H60*B*⋯O4^ii^	0.84 (2)	2.47 (3)	2.993 (2)	122 (3)
C6—H6*B*⋯O6^i^	0.98	2.54	3.411 (3)	148
C6—H6*A*⋯Cl3^iv^	0.98	2.91	3.762 (3)	146

Symmetry codes: (i) 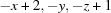; (ii) 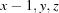; (iii) 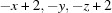; (iv) 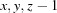.

**Fig. 1.**
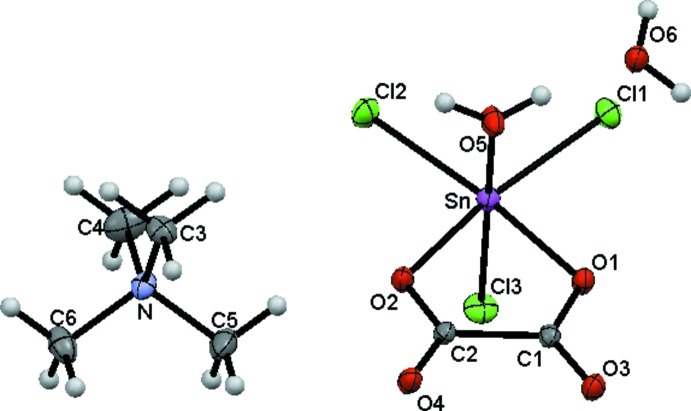
The asymmetric unit showing the numbering scheme. Displacement ellipsoids are drawn at the 50% probability level.

**Fig. 2.**
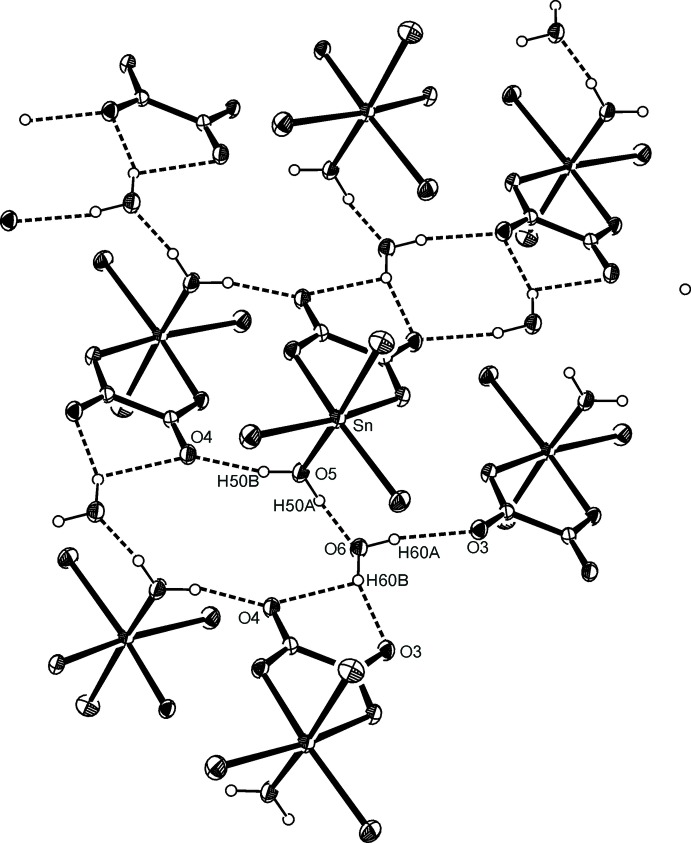
The layered structure of the anions and the lattice water molecule parallel to (010). O—H···O hydrogen bonding interactions are shown as dashed lines.

**Fig. 3.**
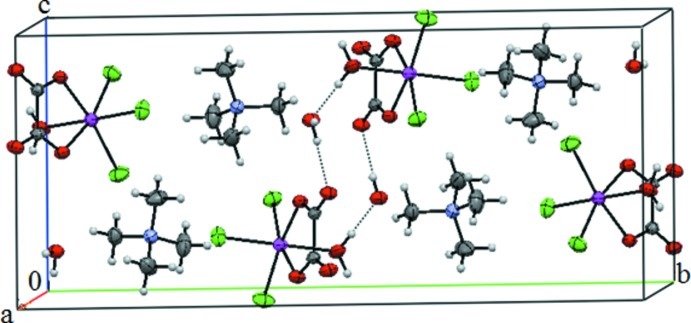
The packing of the structure showing O—H···O hydrogen bonding interactions as dashed lines [C—H···O and C—H···Cl contacts are omitted for clarity].

## supplementary crystallographic information

### Comment

Numerous crystal structures of Sn*X*_4_ adducts (*X* = halogen) containing tin(IV) in an octahedral environment have been reported up to date, e.g. Hausen *et al.* (1986); Koutsantonis *et al.* (2003); Mahon *et al.* (2004); Patt-Siebel *et al.* (1986); Szymanska-Buzar *et al.* (2001); Tudela *et al.* (1986). Our group has previously reported the crystal structure of ((*n*-C_3_H_7_)_2_NH_2_)_2_[Sn(C_2_O_4_)Cl_4_] which contains a chelating oxalate anion, and the environment of tin(IV) being likewise octahedral (Sow *et al.*, 2010). In the context of our search for new Sn*X*_4_ adducts we report here the study of the reaction between ((CH_3_)_4_N)_2_C_2_O_4_^.^2H_2_O and SnCl_4_ which has yielded the title compound, ((CH_3_)_4_N)[Sn(C_2_O_4_)Cl_3_(H_2_O)]^.^H_2_O. While many Sn*X*_4_ adducts have been reported (see above), a complex with a [SnCl_3_]-containing residue is reported here.

The octahedral geometry around the tin(IV) atom is defined by three Cl atoms, two oxygen atoms from the chelating oxalate anion and the oxygen atom of a water molecule (Fig. 1). The two oxygen atoms from the oxalate anion and two of the Cl atoms are in the equatorial plane while the remaining Cl atom and the oxygen atom of the H_2_O molecule are in axial positions.

The [Sn(C_2_O_4_)Cl_3_(H_2_O)]^-^ anions are connected to the lattice water molecule through H—O—H···OH_2_ hydrogen bonds. The water molecule bonded to the tin(IV) atom is also hydrogen-bonded to the O4 atom of a neighbour complex-anion. The lattice water molecule O6 is bonded to O3 and O4 of the same oxalate anion through a bifurcated hydrogen bond and to a O3 atom of a neighbouring oxalate anion, leading to a layered structure extending parallel to (010). The cations are located between the anionic planes (Figs. 2,3). In the crystal packing, C—H···O and C—H···Cl interactions between cations and anions are also observed (Table 1).

The angle O5—Sn—Cl3 [170.75°(5)] deviates from linearity. The two Sn—Cl bond lengths in the equatorial plane are very similar [Sn—Cl2 = 2.3598 (5), Sn—Cl1 = 2.3627 (5) Å], but different from the one *trans* to the water molecule [Sn—Cl3 = 2.3926 (5) Å], pointing to a weak *trans*-effect involving the latter. The Sn—O5 bond of 2.0781 (15) Å involving the water molecule is shorter than the Sn—O bonds distances involving the oxalate anion [Sn—O1 = 2.0980 (13); Sn—O2 = 2.1025 (13) Å], whereby these two last Sn—O distances are very close. The dimensions of Sn—O bonds and Sn—Cl bonds are in the range of Sn—O and Sn—Cl bonds reported for O_2_SnCl_4_ containing adducts with *cis*- or *trans*-geometry (Fernandez *et al.*, 2002; Hazell *et al.*, 1998; Sow *et al.*, 2010).

The C—O distances [O1—C1 = 1.285 (2); O2—C2 = 1.288 (2) Å; O3—C1 = 1.219 (2) Å; O4—C2 = 1.223 (2) Å] are in the typical range of C—O and C═O bonds (Ng & Kumar Das, 1993; Xu *et al.*, 2003).

### Experimental

All chemicals were purchased from Aldrich (Germany) and used without any further purification. ((CH_3_)_4_N)_2_C_2_O_4_^.^2H_2_O has been obtained on allowing ((CH_3_)_4_N)OH as a 20% water solution to react with oxalic acid in a 2:1 ratio. A powder is obtained after evaporation of water at 333 K. On allowing the oxalic acid salt to react with SnCl_4_ in a 1:1 ratio in ethanol, a colorless solution is obtained, which gives, after slow solvent evaporation, crystals suitable for X-ray determination . The reaction equation of the title compound is: ((CH_3_)_4_N)_2_C_2_O_4_^.^2H_2_O + SnCl_4_→ ((CH_3_)_4_N)Cl + ((CH_3_)_4_N)[Sn(C_2_O_4_)Cl_3_H_2_O]^.^H_2_O

### Refinement

Water molecule hydrogen atoms have been located in the difference fourier map and were refined with an idealized bond lenght of 0.85 Å. The other hydrogen atoms have been placed onto calculated position and were refined using a riding model, with C—H distances of 0.98 Å and *U*_iso_(H) = 1.5*U*_eq_(C).

### Crystal data

**Table:**

(C_4_H_12_N)[Sn(C_2_O_4_)Cl_3_(H_2_O)]·H_2_O	*F*(000) = 832
*M**_r_* = 423.24	*D*_x_ = 1.831 Mg m^−^^3^
Monoclinic, *P*2_1_/*n*	Mo *K*α radiation, λ = 0.71073 Å
Hall symbol: -P 2yn	Cell parameters from 29534 reflections
*a* = 7.2458 (1) Å	θ = 2.9–30.0°
*b* = 22.2812 (2) Å	µ = 2.20 mm^−^^1^
*c* = 9.6019 (1) Å	*T* = 150 K
β = 98.015 (1)°	Irregular, colourless
*V* = 1535.04 (3) Å^3^	0.15 × 0.15 × 0.13 mm
*Z* = 4	

### Data collection

**Table:**

Nonius KappaCCD diffractometer	4445 independent reflections
Radiation source: fine-focus sealed tube	3855 reflections with *I* > 2σ(*I*)
Graphite monochromator	*R*_int_ = 0.042
461 1.3 degree images with ω scans	θ_max_ = 30.0°, θ_min_ = 4.2°
Absorption correction: multi-scan (*SORTAV*; Blessing, 1995)	*h* = −10→10
*T*_min_ = 0.734, *T*_max_ = 0.763	*k* = −28→31
35849 measured reflections	*l* = −13→13

### Refinement

**Table:**

Refinement on *F*^2^	Secondary atom site location: difference Fourier map
Least-squares matrix: full	Hydrogen site location: inferred from neighbouring sites
*R*[*F*^2^ > 2σ(*F*^2^)] = 0.026	H atoms treated by a mixture of independent and constrained refinement
*wR*(*F*^2^) = 0.062	*w* = 1/[σ^2^(*F*_o_^2^) + (0.0322*P*)^2^ + 0.5616*P*] where *P* = (*F*_o_^2^ + 2*F*_c_^2^)/3
*S* = 1.08	(Δ/σ)_max_ = 0.001
4445 reflections	Δρ_max_ = 0.92 e Å^−^^3^
175 parameters	Δρ_min_ = −0.79 e Å^−^^3^
4 restraints	Extinction correction: *SHELXL97* (Sheldrick, 2008), Fc^*^=kFc[1+0.001xFc^2^λ^3^/sin(2θ)]^-1/4^
Primary atom site location: structure-invariant direct methods	Extinction coefficient: 0.0124 (5)

### Special details

**Table:**

Geometry. All e.s.d.'s (except the e.s.d. in the dihedral angle between two l.s. planes) are estimated using the full covariance matrix. The cell e.s.d.'s are taken into account individually in the estimation of e.s.d.'s in distances, angles and torsion angles; correlations between e.s.d.'s in cell parameters are only used when they are defined by crystal symmetry. An approximate (isotropic) treatment of cell e.s.d.'s is used for estimating e.s.d.'s involving l.s. planes.
Refinement. Refinement of *F*^2^ against ALL reflections. The weighted *R*-factor *wR* and goodness of fit *S* are based on *F*^2^, conventional *R*-factors *R* are based on *F*, with *F* set to zero for negative *F*^2^. The threshold expression of *F*^2^ > σ(*F*^2^) is used only for calculating *R*-factors(gt) *etc*. and is not relevant to the choice of reflections for refinement. *R*-factors based on *F*^2^ are statistically about twice as large as those based on *F*, and *R*- factors based on ALL data will be even larger.

### Fractional atomic coordinates and isotropic or equivalent isotropic displacement parameters (Å^2^)

**Table:**

	*x*	*y*	*z*	*U*_iso_*/*U*_eq_	
Sn	0.834510 (17)	0.112222 (6)	0.679281 (12)	0.02693 (6)	
Cl1	0.61541 (8)	0.13476 (3)	0.83233 (5)	0.03867 (12)	
Cl2	0.66559 (8)	0.14793 (3)	0.46760 (5)	0.04369 (14)	
Cl3	1.01222 (8)	0.20243 (2)	0.72319 (6)	0.04152 (13)	
O5	0.7216 (2)	0.02693 (7)	0.64413 (15)	0.0372 (3)	
O1	1.00588 (18)	0.07154 (6)	0.84708 (13)	0.0278 (3)	
O3	1.2565 (2)	0.01364 (6)	0.89271 (13)	0.0310 (3)	
O4	1.28776 (19)	0.01308 (7)	0.61246 (13)	0.0323 (3)	
O2	1.04412 (18)	0.07556 (6)	0.57415 (13)	0.0285 (3)	
O6	0.5915 (2)	−0.03357 (7)	0.82856 (15)	0.0320 (3)	
N	1.0670 (2)	0.16827 (7)	0.20003 (17)	0.0298 (3)	
C1	1.1444 (2)	0.04194 (8)	0.81171 (17)	0.0241 (3)	
C2	1.1635 (3)	0.04294 (8)	0.65224 (18)	0.0249 (3)	
C3	0.9820 (3)	0.10701 (9)	0.1966 (3)	0.0370 (5)	
H3A	0.8911	0.1053	0.2632	0.055*	
H3B	1.0798	0.0771	0.2228	0.055*	
H3C	0.9192	0.0985	0.1015	0.055*	
C4	0.9184 (4)	0.21327 (11)	0.1570 (3)	0.0561 (7)	
H4A	0.8558	0.2036	0.0624	0.084*	
H4B	0.9739	0.2534	0.1566	0.084*	
H4C	0.8274	0.2125	0.2235	0.084*	
C5	1.1603 (4)	0.18245 (13)	0.3445 (2)	0.0500 (6)	
H5A	1.2132	0.2230	0.3458	0.075*	
H5B	1.2601	0.1533	0.3721	0.075*	
H5C	1.0689	0.1804	0.4106	0.075*	
C6	1.2081 (4)	0.17066 (11)	0.0997 (3)	0.0491 (6)	
H6A	1.1482	0.1599	0.0051	0.074*	
H6B	1.3090	0.1423	0.1300	0.074*	
H6C	1.2592	0.2113	0.0984	0.074*	
H50B	0.703 (4)	0.0121 (13)	0.562 (2)	0.058 (8)*	
H60B	0.481 (3)	−0.0227 (14)	0.829 (3)	0.057 (9)*	
H60A	0.647 (4)	−0.0270 (13)	0.909 (2)	0.053 (8)*	
H50A	0.668 (4)	0.0068 (12)	0.704 (3)	0.059 (9)*	

### Atomic displacement parameters (Å^2^)

**Table:**

	*U*^11^	*U*^22^	*U*^33^	*U*^12^	*U*^13^	*U*^23^
Sn	0.02791 (9)	0.02922 (8)	0.02369 (8)	0.00383 (4)	0.00368 (5)	0.00042 (4)
Cl1	0.0382 (3)	0.0436 (3)	0.0363 (3)	0.0062 (2)	0.0124 (2)	−0.0069 (2)
Cl2	0.0376 (3)	0.0596 (3)	0.0325 (3)	0.0146 (2)	0.0002 (2)	0.0094 (2)
Cl3	0.0423 (3)	0.0295 (2)	0.0520 (3)	−0.0019 (2)	0.0039 (2)	0.0029 (2)
O5	0.0468 (9)	0.0414 (8)	0.0256 (7)	−0.0136 (7)	0.0122 (6)	−0.0087 (6)
O1	0.0321 (7)	0.0308 (6)	0.0208 (6)	0.0045 (5)	0.0048 (5)	−0.0001 (5)
O3	0.0316 (7)	0.0376 (7)	0.0230 (6)	0.0047 (6)	0.0014 (5)	0.0034 (5)
O4	0.0291 (7)	0.0442 (8)	0.0230 (6)	0.0069 (6)	0.0018 (5)	−0.0051 (5)
O2	0.0287 (7)	0.0362 (7)	0.0209 (6)	0.0051 (5)	0.0040 (5)	0.0033 (5)
O6	0.0323 (8)	0.0396 (8)	0.0239 (7)	0.0062 (6)	0.0031 (6)	0.0003 (5)
N	0.0361 (9)	0.0254 (7)	0.0272 (8)	−0.0021 (6)	0.0018 (6)	−0.0007 (6)
C1	0.0268 (9)	0.0247 (8)	0.0205 (8)	−0.0031 (6)	0.0024 (7)	−0.0013 (6)
C2	0.0260 (9)	0.0281 (8)	0.0199 (8)	−0.0025 (7)	0.0014 (6)	−0.0014 (6)
C3	0.0409 (12)	0.0269 (9)	0.0445 (12)	−0.0048 (8)	0.0106 (10)	−0.0014 (8)
C4	0.0539 (15)	0.0325 (12)	0.0774 (19)	0.0084 (10)	−0.0061 (13)	0.0040 (11)
C5	0.0551 (15)	0.0598 (15)	0.0319 (11)	−0.0207 (12)	−0.0052 (10)	0.0029 (10)
C6	0.0646 (16)	0.0399 (12)	0.0477 (13)	−0.0152 (11)	0.0249 (12)	−0.0067 (10)

### Geometric parameters (Å, º)

**Table:**

Sn—O5	2.0781 (15)	N—C3	1.496 (2)
Sn—O1	2.0980 (13)	N—C6	1.500 (3)
Sn—O2	2.1025 (13)	C1—C2	1.557 (2)
Sn—Cl2	2.3598 (5)	C3—H3A	0.9800
Sn—Cl1	2.3627 (5)	C3—H3B	0.9800
Sn—Cl3	2.3926 (5)	C3—H3C	0.9800
O5—H50B	0.850 (17)	C4—H4A	0.9800
O5—H50A	0.859 (17)	C4—H4B	0.9800
O1—C1	1.285 (2)	C4—H4C	0.9800
O3—C1	1.219 (2)	C5—H5A	0.9800
O4—C2	1.223 (2)	C5—H5B	0.9800
O2—C2	1.288 (2)	C5—H5C	0.9800
O6—H60B	0.836 (17)	C6—H6A	0.9800
O6—H60A	0.836 (17)	C6—H6B	0.9800
N—C4	1.488 (3)	C6—H6C	0.9800
N—C5	1.490 (3)		
			
O5—Sn—O1	84.67 (6)	O1—C1—C2	115.63 (15)
O5—Sn—O2	82.02 (6)	O4—C2—O2	126.11 (16)
O1—Sn—O2	79.11 (5)	O4—C2—C1	118.03 (16)
O5—Sn—Cl2	91.33 (5)	O2—C2—C1	115.85 (15)
O1—Sn—Cl2	170.93 (4)	N—C3—H3A	109.5
O2—Sn—Cl2	92.30 (4)	N—C3—H3B	109.5
O5—Sn—Cl1	90.68 (4)	H3A—C3—H3B	109.5
O1—Sn—Cl1	89.50 (4)	N—C3—H3C	109.5
O2—Sn—Cl1	166.95 (4)	H3A—C3—H3C	109.5
Cl2—Sn—Cl1	98.70 (2)	H3B—C3—H3C	109.5
O5—Sn—Cl3	170.75 (5)	N—C4—H4A	109.5
O1—Sn—Cl3	88.93 (4)	N—C4—H4B	109.5
O2—Sn—Cl3	90.23 (4)	H4A—C4—H4B	109.5
Cl2—Sn—Cl3	94.03 (2)	N—C4—H4C	109.5
Cl1—Sn—Cl3	95.95 (2)	H4A—C4—H4C	109.5
Sn—O5—H50B	121 (2)	H4B—C4—H4C	109.5
Sn—O5—H50A	125 (2)	N—C5—H5A	109.5
H50B—O5—H50A	113 (3)	N—C5—H5B	109.5
C1—O1—Sn	114.77 (11)	H5A—C5—H5B	109.5
C2—O2—Sn	114.29 (11)	N—C5—H5C	109.5
H60B—O6—H60A	107 (3)	H5A—C5—H5C	109.5
C4—N—C5	109.4 (2)	H5B—C5—H5C	109.5
C4—N—C3	109.18 (18)	N—C6—H6A	109.5
C5—N—C3	110.24 (17)	N—C6—H6B	109.5
C4—N—C6	109.2 (2)	H6A—C6—H6B	109.5
C5—N—C6	109.25 (18)	N—C6—H6C	109.5
C3—N—C6	109.49 (16)	H6A—C6—H6C	109.5
O3—C1—O1	124.90 (16)	H6B—C6—H6C	109.5
O3—C1—C2	119.47 (16)		

### Hydrogen-bond geometry (Å, º)

**Table:**

*D*—H···*A*	*D*—H	H···*A*	*D*···*A*	*D*—H···*A*
O5—H50*A*···O6	0.86 (2)	1.66 (2)	2.511 (2)	173 (3)
O5—H50*B*···O4^i^	0.85 (2)	1.78 (2)	2.6120 (19)	168 (3)
O6—H60*B*···O3^ii^	0.84 (2)	1.99 (2)	2.792 (2)	160 (3)
O6—H60*A*···O3^iii^	0.84 (2)	1.95 (2)	2.7840 (19)	172 (3)
O6—H60*B*···O4^ii^	0.84 (2)	2.47 (3)	2.993 (2)	122 (3)
C6—H6*B*···O6^i^	0.98	2.54	3.411 (3)	148
C6—H6*A*···Cl3^iv^	0.98	2.91	3.762 (3)	146

Symmetry codes: (i) −*x*+2, −*y*, −*z*+1; (ii) *x*−1, *y*, *z*; (iii) −*x*+2, −*y*, −*z*+2; (iv) *x*, *y*, *z*−1.
